# Effects of Artificially Modified Microbial Communities on the Root Growth and Development of Tall Fescue in Nutrient-Poor Rubble Soil

**DOI:** 10.3390/plants13233307

**Published:** 2024-11-25

**Authors:** Zhengyu Luo, Hongrui Han, Hui Yao, Guoru Yan, Jinxin Bai, Lihao Shi, Xiangjun Pei, Jingji Li, Qiang Li

**Affiliations:** 1 College of Environmental and Civil Engineering, Chengdu University of Technology, Chengdu 610059, China; luozhengyu15@cdut.edu.cn; 2 School of Ecology and Environment, Chengdu University of Technology, Chengdu 610059, China; han032311@163.com (H.H.); 13330682689@163.com (H.Y.); ygr4399@163.com (G.Y.); 18636830676@163.com (J.B.); slh1204@163.com (L.S.); 3 Key Laboratory of Synergistic Control and Joint Remediation of Soil and Water Pollution of National Environmental Protection, Chengdu 610059, China; 4 Xinjiang Uygur Autonomous Region Geology and Mineral Exploration and Development Bureau, Urumqi 830052, China; 18583388036@163.com

**Keywords:** tall fescue, microbiota, root structure, anatomical structure

## Abstract

The granite rubble soil produced through excavation during construction is nutrient-poor and has a simplified microbial community, making it difficult for plants to grow and increasing the challenges of ecological restoration. Recent studies have demonstrated that microbial inoculants significantly promote plant growth and are considered a potential factor influencing root development. Microorganisms influence root development either directly or indirectly, forming beneficial symbiotic relationships with plant roots. However, the mechanisms by which microorganisms affect root development and root anatomy, as well as the dynamics of soil microbial communities following the artificial application of microbial inoculants, remain unclear. This experiment utilized granite rubble soil from construction excavation in a pot trial, implementing five different treatment methods. After the fast-growing grass species tall fescue (*Festuca arundinacea*) was planted, four growth-promoting microbial inoculants—*Bacillus subtilis* (K), *Bacillus amyloliquefaciens* (JD), *Aspergillus niger* (H), and *Trichoderma harzianum* (HC)—were applied to the soil in the pots. These treatments were compared with a control group (CK) that received no microbial inoculant. At 120 days of plant growth, the composition of the soil microbial community, biomass, root structure, and root anatomy were measured for each treatment group. This analysis aimed to explore the effects of different microbial treatments on the microbial communities and root development of *Festuca arundinacea* root soil. The study found that the addition of microbial inoculants reduced the number of microbial operational taxonomic units (OTUs) of bacteria and fungi in the soil, affecting both the marker species and their abundance at the phylum level. Additionally, microbial inoculants promoted the development of the tall fescue root structure, increasing metrics such as the total root length, root surface area, root volume, and root-to-shoot ratio per plant. Redundancy analysis (RDA) revealed that the area ratios of various components in the root anatomy of tall fescue’s primary roots, such as the root cortex area, stele area, and the number of lateral roots, were influenced by *Proteobacteria*. *Mortierellomycota* was found to affect the root epidermis area.

## 1. Introduction

Granite rubble soil generated through excavation in engineering construction is a unique soil type characterized by high porosity, poor stability, low water retention capacity, nutrient deficiency, and a simple microbial community structure. These unfavorable conditions make it challenging for plants to grow in such soils [1]. In response to this adversity, plants must adapt by altering their physiological and structural traits to absorb more nutrients and water to sustain their growth, thereby mitigating the negative impacts of the environment [2].

Roots are essential organs for plant absorption and metabolism, directly influencing a plant’s ability to uptake water and nutrients [3,4]. Additionally, plant roots can enhance soil nutrient content by releasing exudates that help decompose otherwise insoluble elements in the soil [5]. Given their direct contact with the soil, roots are highly sensitive to environmental factors [6]. Root architecture is a crucial agronomic and ecological indicator, as its characteristics directly reflect root growth status. Under stress from biotic or abiotic factors, root architecture often adapts first in a direction that favors plant growth [7,8]. Thus, a well-developed root architecture is vital for both plant growth and ecological stability. The anatomical structure of roots serves as a direct indicator of the degree of root development, which is closely tied to plant physiological functions [9]. Research has shown that factors such as the degree of lignification and the development of vascular tissues in root anatomy can significantly influence a plant’s ability to withstand environmental changes.

Tall fescue (*Festuca arundinacea*) is a perennial herbaceous plant of the *Poaceae* family, characterized by a root system with numerous fine lateral roots [10,11]. This specialized root structure enhances its ability to absorb water and nutrients while stabilizing the soil. Due to the unique properties of its root system, tall fescue plays a crucial role under adverse conditions and is widely used in China as a remedial plant for ecological restoration in such environments [12]. It has been demonstrated that tall fescue can degrade heavy metal pollutants and reinforce soil through its root system [13]. However, research on the root architecture of tall fescue in nutrient-deficient granite rubble soil remains limited.

Soil microorganisms are a critical component of the soil ecosystem and play a significant role within the broader ecological system [14,15]. For instance, *Bacillus* species, which are plant growth-promoting rhizobacteria (PGPR), can secrete growth hormones that increase root length and the number of lateral roots, thereby enhancing the plant’s ability to absorb soil nutrients and improving its stress resistance [16]. Arbuscular mycorrhizal fungi (AMF) can form a mycelial network with plant roots, significantly boosting the root system’s nutrient absorption capacity, which in turn promotes plant growth and survival [17,18]. Studies have demonstrated that the application of single or composite microorganisms to soil can alter plant root structures, such as promoting lateral root formation, thereby increasing plant adaptability [19]. However, current research on the effects of microorganisms on plant root systems has mainly focused on general soil conditions, with relatively scarce studies in nutrient-poor granite rubble soil. Although it has been established that microorganisms have a significant impact on plant root structure under ordinary soil conditions, research remains limited in the context of granite rubble soil, particularly concerning tall fescue. Whether the targeted application of microbial agents to granite rubble soil can induce the formation of functional microbial communities necessary for promoting the growth and development of tall fescue root structures remains to be investigated. Additionally, it is still necessary to explore whether microorganisms can influence the anatomical structure of the primary root cross-section of tall fescue while altering the root system structure.

In summary, in this study, we conducted an indoor pot experiment using nutrient-poor granite rubble soil to cultivate tall fescue, a species known for its extensive root system and strong adaptability. Four beneficial microorganisms—*Bacillus subtilis*, *Bacillus amyloliquefaciens*, *Aspergillus niger*, and *Trichoderma harzianum*—were applied, each known for their growth-promoting and nutrient-decomposing properties. This study aimed to investigate the changes in the microbial community within the granite rubble soil under the influence of these four microorganisms. Additionally, it explored the effects of microbial community changes on the growth, development, and anatomical structure of tall fescue roots. This research enriches our understanding of how microorganisms influence the growth and development of tall fescue root architecture under gravel soil conditions. It provides microbial solutions for improving crop growth in barren soils and offers theoretical support for plant growth in poor soil conditions.

## 2. Results

### 2.1. Changes in the Microbial Community of Tall Fescue Root Soil

(1) Dynamics of the fungal community

**Figure 1:**
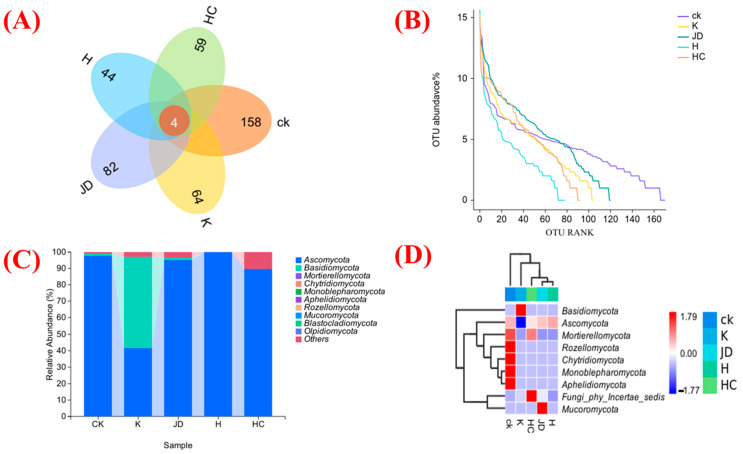
Fungal OTU Wayne plot (**A**), fungal abundance curves (**B**), root soil microbial community composition at the fungal phylum level (**C**), and species composition plot at the fungal phylum level (**D**) for tall fescue root soil fungi under fungus addition treatments. CK indicates the blank treatment; K indicates the *Bacillus subtilis* application; JD indicates the *Bacillus amyloliquefaciens* application; H indicates the *Aspergillus niger* application; and HC indicates the *Trichoderma harzianum* application.

The soil fungi under different treatments were analyzed for their β diversity to characterize their structural features. The operational taxonomic unit (OTU) numbers in the Venn diagram show that four OTUs of the fungal community were common to all samples (Figure 1A). The highest number of OTUs was unique to the rhizosphere soil under the CK treatment (158), and the lowest was unique to the soil to which *Aspergillus niger* was applied (44). Fungal abundance classes were compared in the soil under different treatments using abundance class curve analysis (Figure 1B). The abundance values were high in the native residue soil. The cause of this phenomenon may be the artificial addition of microbial solutions to the soil, which caused a directional change in the microbial species in the soil, and thus, a decrease in the abundance of microorganisms in the soil under the application of the solution treatment.

The fungal abundance in the rhizosphere soil under different treatments was statistically analyzed (Figure 1C). The dominant fungal phylum was Basidiomycota under the K treatment, and the dominant species group at the fungal community phylum level was Ascomycota under the CK and other treatments. The fungal communities in the rhizosphere soils under different microbial liquid treatments were analyzed for marker species. The relative abundances of the fungal phyla that ranked among the top 20 in the soils of different sub-locations are shown in Figure 1D. The CK treatment group had the highest relative abundances of Rozellomycota, Chytridiomycota, Monoblepharomycota, and Aphelidiomycota, and the treatment with *Bacillus subtilis* had a high relative abundance of Basidiomycota. Fungi phy Incertae sedis had a high relative abundance in treatments applying *Trichoderma harzianum*, Mucoromycota had a high relative abundance under treatments with *Bacillus amyloliquefaciens*, and Ascomycota had a high relative abundance under treatments with *Aspergillus niger*, but it was not significant.

(2) Dynamics of the bacterial community

**Figure 2:**
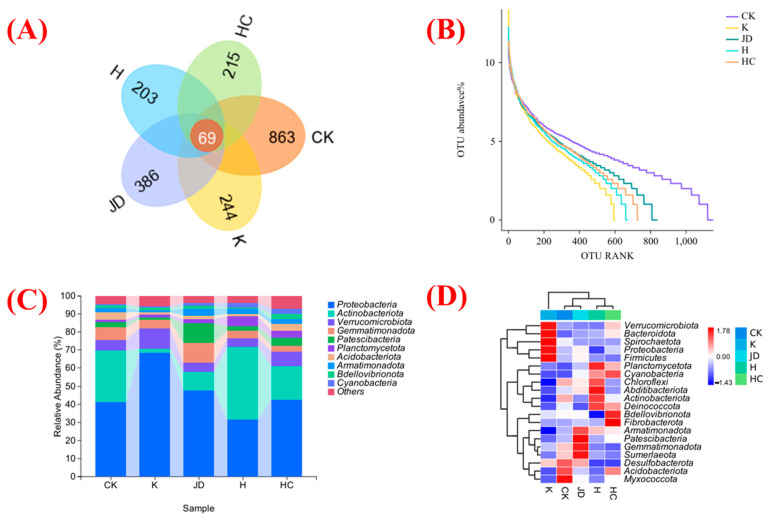
Bacterial OTU Wayne plot (**A**), bacterial abundance curves (**B**), root soil microbial community composition at the bacterial phylum level (**C**), and species composition plot at the bacterial phylum level (**D**) in the root soil of tall fescue under bacterial addition treatments. CK indicates the blank treatment; K indicates the *Bacillus subtilis* application; JD indicates the *Bacillus amyloliquefaciens* application; H indicates the *Aspergillus niger* application; and HC indicates the *Trichoderma harzianum* application.

The soil bacteria under different treatments were analyzed for their β diversity to characterize their structural features. The operational taxonomic unit (OTU) numbers in the Venn diagram show that 69 OTUs of the bacterial community were common to all samples (Figure 2A). The highest number of OTUs unique to the rhizosphere soil was found under the CK treatment (863), whereas the lowest number of OTUs unique to the rhizosphere soil was found under the H treatment (202). The soil bacterial abundance classes of the different treatments were compared using abundance rank curve analysis (Figure 2B). The results indicate that a dominant community suddenly emerged under the bacterial addition treatment, thus reducing the overall species abundance. The bacterial abundance in the rhizosphere soil was statistically analyzed under different treatments (Figure 2C). The results show that Proteobacteria was the dominant species group, followed by Actinobacteriota. The application of *Bacillus subtilis* resulted in Proteobacteria as the dominant population; Actinobacteriota had low abundance, and Verrucomicrobiota had increased abundance. Proteobacteria was also the dominant population under *Bacillus amyloliquefaciens* application, followed by Actinobacteriota, but the abundance of Actinobacteriota was higher than that of Patescibacteria and Gemmatimonadota.

The bacterial community in the rhizosphere soil under treatment was also analyzed for marker species. The relative abundances of the top 20 bacterial phyla in different same-site soils are shown in Figure 2D. Different treatments affected the species and abundances of marker species of soil microbial communities at the phylum level, with a high relative abundance of Myxococcota and Acidobacteriota in the rhizosphere soils under the CK treatment, a high relative abundance of Bacteroidota and Spirochaetota in the rhizosphere soils under the *Bacillus subtilis* treatment, a high relative abundance of Patescibacteria under the *Bacillus amyloliquefaciens* treatment, a high relative abundance of Abditibacteriota under the *Aspergillus niger* treatment, and a high relative abundance of Fibrobacterota under the *Trichoderma harzianum* treatment.

### 2.2. Changes in the Biomass of Tall Fescue

**Figure 3:**
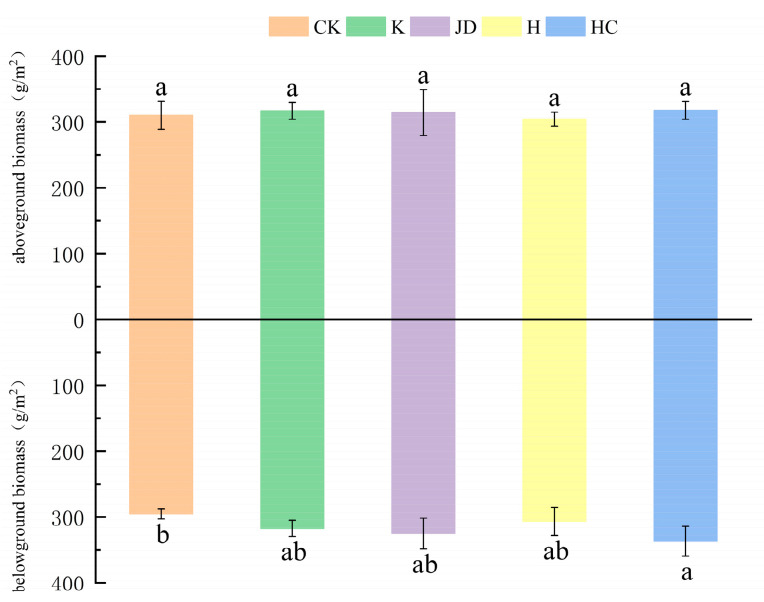
Aboveground and belowground biomasses of tall fescue under the added microbial liquid treatments. Note: Different lowercase letters represent significant differences among the different treatments for the same index (*p* < 0.05). CK indicates the blank treatment; K indicates the *Bacillus subtilis* application; JD indicates the *Bacillus amyloliquefaciens* application; H indicates the *Aspergillus niger* application; and HC indicates the *Trichoderma harzianum* application.

**Figure 4:**
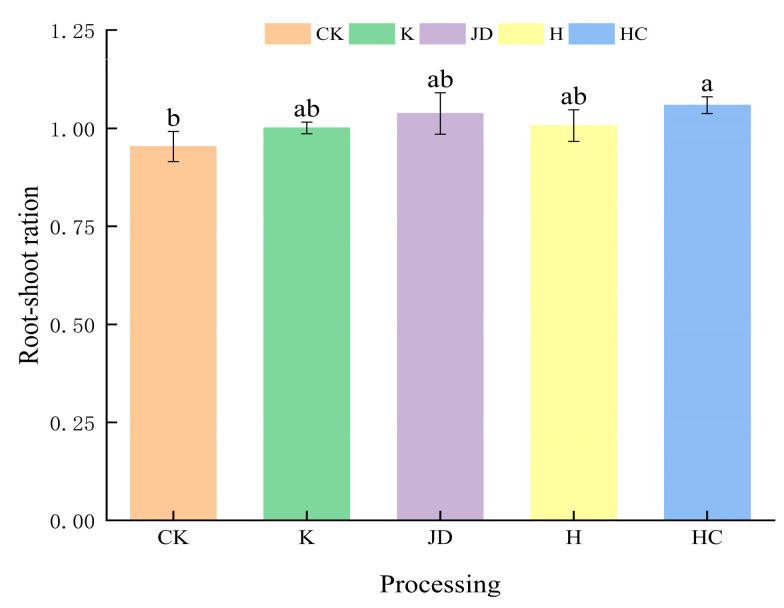
Root–shoot ratio of tall fescue under the added microbial liquid treatments. Note: Different lowercase letters represent significant differences among the different treatments for the same index (*p* < 0.05). CK indicates the blank treatment; K indicates the *Bacillus subtilis* application; JD indicates the *Bacillus amyloliquefaciens* application; H indicates the *Aspergillus niger* application; and HC indicates the *Trichoderma harzianum* application.

As shown in Figure 3 and Figure 4, the aboveground and belowground biomass of tall fescue were affected by the application of microbial agents. The belowground biomasses of the plants treated with microbial liquid increased to different degrees, with that of the HC treatment being significantly higher than that of the CK treatment, while the results of the other treatments were not significant. The aboveground biomasses were greater than the blank treatments in all of the spiked treatments except for the treatment where H was applied. The changes in biomass were accompanied by changes in the root–shoot ratio under the applied microbial solution treatment. As shown in Figure 4, the highest root–shoot ratio was observed in the treatment with HC, in which it increased by 10.5% compared to the blank treatment (*p* < 0.05), followed by the JD application, in which it increased by 8.4% compared to the blank treatment (*p* > 0.05). The growth rates of the root–shoot ratio were similar in the K and H applications, at 4.9 and 5.5% (*p* > 0.05), respectively.

### 2.3. Effects of Microorganisms on the Root Morphology of Tall Fescue

**Figure 5:**
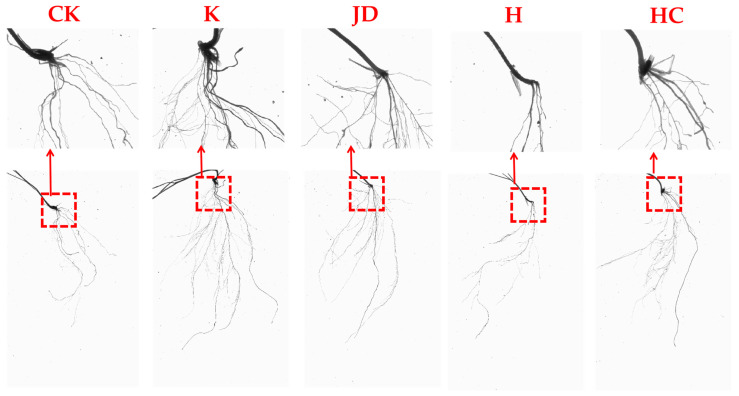
Root system structure of tall fescue under different treatments.

Significant changes in the plant root structure were observed under microbial liquid application (Figure 5), with an increase in the fibrous roots of the plants under the influence of microorganisms. Overall, the morphology of the plant root system under each treatment with the addition of microorganisms was better than that of the plant root system under the blank treatment, except for the treatment with H application (Table 1). The total root length, total root surface area, number of root branches, and number of root forks of the root system of a single tall fescue plant were not significantly different among the treatments (*p* > 0.05). There were significant differences (*p* < 0.05) in the root system volumes of the tall fescue plants between the K and H applications.

Pearson’s correlations among six root conformation parameters were analyzed (Table 2), and highly significant correlations (*p* < 0.01) were found among the following five indicators: total root length, total root surface area, total root volume, number of root tips, and number of root branches. The mean root diameter showed a significant negative correlation trend with the total root length (*p* < 0.05) and a negative correlation with the total root surface area, total root volume, number of root tips, and number of root branches (*p* > 0.05).

The lower part of the image illustrates the root growth of tall fescue under various treatments, while the upper part offers a magnified view of the plant root system to enhance the reader’s to observe structural changes.

### 2.4. Changes in the Transverse Anatomical Structure of Tall Fescue Main Roots

**Figure 6:**
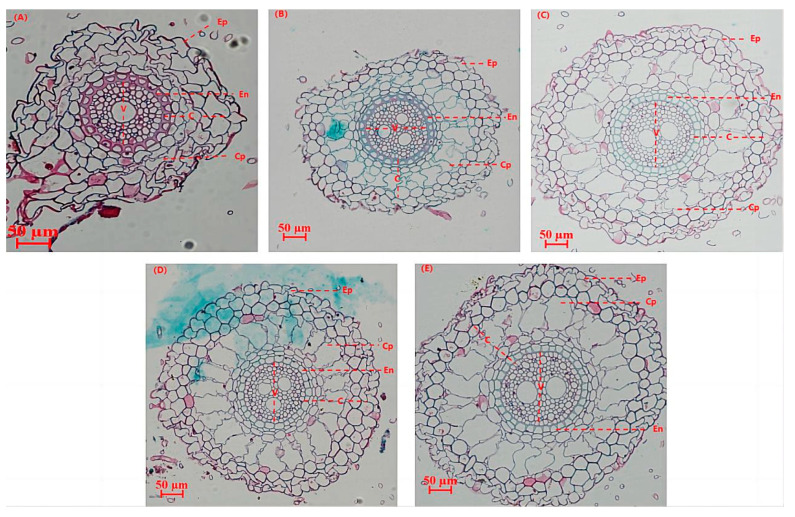
The transverse anatomical structure of the primary root of tall fescue after the addition of microbial liquid treatments. Note: (**A**) blank treatment; (**B**) application of *Bacillus subtilis*; (**C**) application of *Bacillus amyloliquefaciens*; (**D**) application of *Aspergillus niger*; and (**E**) application of *Trichoderma harzianum*. Ep: epidermis; Cp: cortical parenchyma; En: endodermis; C: cortex; V: vascular.

As shown in Figure 6, the root structure of the tall fescue in the untreated group differed significantly from that in the microbial treatment group, with the latter exhibiting more advanced root development. The root system of the plants treated with microbial liquid solution was better developed than that of the plants treated without microbial liquid solution. The root epidermal cells showed an irregular multilayered cell structure under the non-microbial liquid treatment. Under the influence of microbial liquid, the root epidermis had a regular shape and showed a single- or double-layered cell structure similar to a rectangle. The outer cortex of the microbial liquid solution groups all showed a single layer of closely arranged cells, while the non-microbial liquid groups did not show a clear pattern. No embolic structures were found in the cell wall of the outer cortex under all treatment groups. The thickness of the cortical thin-walled tissue was narrow in the non-microbial liquid treatment, and the cells were arranged in a disordered manner. The cortical thin-walled cells under the microbial liquid treatment were arranged in a regular and orderly manner with “radial” cellular interstices. The cell walls of the endodermis under each treatment were “horseshoe-shaped” and thickened. The mid-column xylem was well developed, and the conduits were clearly visible in all treatments, but the number of conduits was lower in the non-microbial liquid treatment than in the microbial liquid treatment.

**Figure 7:**
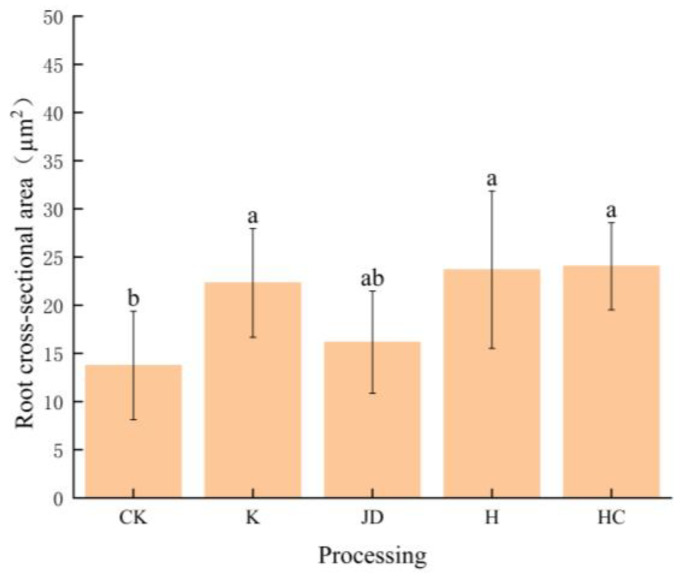
Area of the transverse anatomical structure of the main root of tall fescue after the addition of bacteria treatments. Note: Different lowercase letters represent significant differences among the different treatments for the same index (*p* < 0.05).

The main root cross-sectional area was (13.76 ± 5.72) × 10^4^ µm^2^ in the non-microbial liquid treatment group, while the lower root cross-sectional area was (22.32 ± 5.65) × 10^4^ µm^2^ in the K-treated group, (16.16 ± 5.30) × 10^4^ µm^2^ in the JD-treated group, (20.63 ± 9.83) × 10^4^ µm^2^ in the H-treated group, and (24.05 ± 4.51) × 10^4^ µm^2^ in the H-treated group. No significant difference was observed in root cross-sectional area between the control group and the *Bacillus amyloliquefaciens* treatment group. However, the CK treatment showed significant differences compared to all the other microbial treatment groups (*p* < 0.05) (Figure 7).

**Figure 8:**
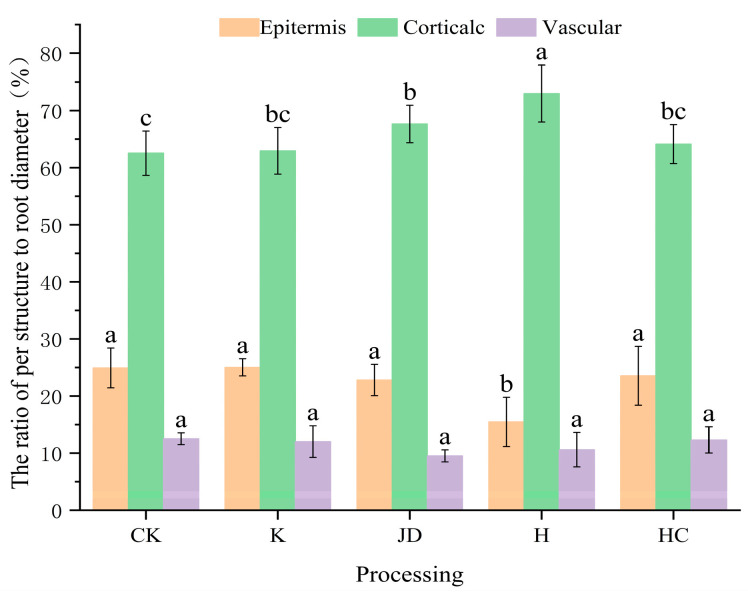
The transverse anatomical structure of the main root of tall fescue after the addition of bacteria treatments, represented as the proportions of the diameter occupied by each treatment. Note: Different lowercase letters represent significant differences among the different treatments for the same index (*p* < 0.05).

Microorganisms affected the percentage of the root cross-sectional structure area (Figure 8). Compared to the blank treatment group, *Bacillus subtilis* increased the percentage of the root epidermal area (*p* > 0.05). The percentage of the root epidermal area was slightly reduced by the action of JD and HC, but the difference was not significant (*p* > 0.05). H significantly reduced the percentage of the epidermal area (*p* < 0.05), and the percentage of the area of the mesocolumn was reduced by all additive bacterial treatments (*p* > 0.05). The cortical areas under the influence of the H and JD treatments were significantly greater than that of the control (*p* < 0.05).The occupied diameter ratio of the endodermis in the CK treatment was higher than in the other treatments, though the difference was not statistically significant (*p* > 0.05).

In conclusion, the root transverse areas under each treatment were in the order HC > K > H > JD > CK; the percentages of epidermis were in the order K > CK > HC > JD > H; the percentages of cortex were in the order H > JD > K > HC > CK; and the percentages of endodermis were in the order CK > HC > K > H > JD.

## 3. Materials and Methods

### 3.1. Materials

**Figure 9:**
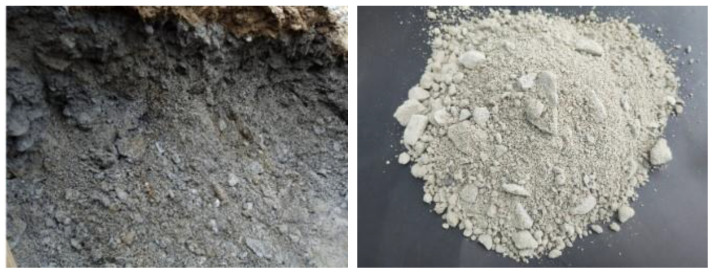
Experimental granite rubble soil.

The growth experiments were carried out indoors in a constant-temperature greenhouse at 18–25 °C with 30% humidity. The soil used in the experiment was poor silt sandy soil (Table 3, Figure 9). The tall fescue seeds used in the experiment were purchased from Chengdu Grassland Science Research Institute, Chengdu, Sichuan, China, with a 93% germination rate and good resistance. The microbial agents used in the experiment, *Bacillus subtilis* (ATCC6633), *Bacillus amyloliquefaciens* (AS1.836), *Aspergillus niger* (ATCC16404), and *Trichoderma harzianum* (ACCC30371), were purchased from the Shanghai Conservation Microbiology Center, Shanghai, China. This experiment simulated the natural environment of the microbial community in gravel soil, so the soil was not sterilized.

In this paper, CK represents the control treatment, K represents the treatment with *Bacillus subtilis*, JD represents the treatment with *Bacillus amyloliquefaciens*, H represents the treatment with *Aspergillus niger*, and HC represents the treatment with *Trichoderma harzianum*.

### 3.2. Experimental Design

The experiment was conducted in indoor pots using round plastic flowerpots (20 cm height and 15 cm diameter). Each pot was quantitatively filled with 4 kg of soil, and tall fescue seeds were sown at a dosage of 0.8 g/ flowerpot. There were five treatments, and 10 mL (10^8^ cfu/mL) of microbial liquid medium was applied to each pot for each treatment. For each treatment, five pots were planted, for a total of twenty-five pots. The same dose of microbial liquid medium was applied every 30 days after planting, and 100 mL of ultrapure water was added daily.

### 3.3. Microbial Purification and Application of Bacterial Fluids

Each of the four microbial strains was used to inoculate LB liquid medium (Luria–Bertani Broth, Maclean’s Biochemical Technology, Shanghai, China) and placed on a shaker for 2 days (180 r/min, 28 °C). It was added to the soil when the bacterial content of the liquid medium reached 10^8^ cfu/mL.

### 3.4. Root Collection and Paraffin Section Preparation

At 120 days after planting, the pots were cut, and the root systems of the plants under different treatments were collected. Single intact plant roots were separated, and the root-adherent soil was removed. The main roots of five plants were selected for each treatment and placed in FAA fixative to produce paraffin sections of the root systems. Then, five plants under different treatments were randomly selected, and the root systems were scanned using a desktop scanner.

The roots were removed from the fixative, and root segments of approximately 1 cm in length at the root tip were individually intercepted. They were washed with 50% ethanol solution, then dehydrated with tert-butanol solution (20, 35, 55, and 75% in a concentration gradient) for 1.5 h at each gradient. The materials were infiltrated with a tert-butanol/pure paraffin (1:1) solution for 3 h and then pure paraffin for 3 days. The material was embedded using a KD-BM II paraffin embedding machine and sliced using a LEICARM 2145 manual rotary slicer at thicknesses of 10–12 μm. Finally, the slices were dried on a 40 °C roaster, stained using fenugreek fixed green staining, and sealed with tree resin. Five primary roots of the treated plants were selected to make root paraffin transverse sections. One complete transverse section was cut from each root, and three replicates were prepared to produce 5 × 5 × 3 = 75 sections.

The root system images were analyzed using Win-RHIZO root system analysis software to obtain the indicators of the total root length, total root surface area, total root volume, average root diameter, number of root tips, and number of root branches of the root system of a single tall fescue plant. The transverse anatomical structures of the root system of tall fescue plants under different treatments were observed using a light microscope. The indicators of the transverse area, epidermal area, cortical area, and mid-column area of the main root were measured using Motic Images Plus 2.0 image analysis software, and the ratio of the area of each part of the anatomical structure to the transverse area of the root was calculated.

### 3.5. Determination of Biomass

After the treatments for root analysis were removed, the remaining plants in each pot were mowed flush to the ground to obtain aboveground biomass, baked in an oven at 65 °C until a constant weight was achieved, and weighed. After the aboveground biomass was harvested, the underground root system was rinsed with water and placed in an oven at 65 °C until a constant weight was achieved. The root–crown ratio was calculated as follows: root–crown ratio = root biomass/crown biomass.

### 3.6. Determination of Soil Microbial Community Diversity and Composition

Soil was removed from the plant residues with other impurities and sent in bulk to Shanghai Parsonage Co., Ltd. (Shanghai, China) for microbial sequencing and subsequent analysis. Microbial DNA was extracted using an OmegaDNA kit, and primers 338F (5′-ATCCCTACGGGGAGGCAGCA-3′) and 806R (5′-GACTACHVGGGGTWTCTAAT-3′) were used to analyze the 16S rRNA in the plant rhizosphere bacteria under different treatments. The ITS1(a) region in the fungi from the root soil was amplified using the PCR universal primers ITS5F (GGAAGTAAAAGTCGTAACAAGG) and ITS1R (GCTCCGTTCTTCATCGATGC). The ITS1(a) region was amplified. Microbial bioinformatics analyses were mainly performed using QIIME2, and OTU clustering was performed using Vsearch (v2.13.14).

### 3.7. Data Analysis

The data were organized using Excel, and a two-way analysis of variance was performed using IBM SPSS Statistics26 to detect the differences among the root anatomical structures of the different treatments. Pearson’s correlation analysis was then performed to evaluate the relationships among the root structures, and the results were considered statistically significant at *p* < 0.05. The results were plotted using OriginPro 2021 (9.8.0.200) for mapping. The correlation between the microbial phyla and the plant root traits was analyzed using redundancy analysis (RDA). Microbial data mapping was performed using the platform from Paysono Biotechnology Co. (Pasono Bio-technology, Shanghai, China).

## 4. Discussion

Rhizosphere microorganisms are often referred to as plants’ second genomes, playing a critical role in plant growth and development [20]. In this study, an indoor pot experiment was conducted using tall fescue grown in granite rubble soil derived from tunnel excavation. Microbial inoculants, including *Bacillus subtilis*, *Bacillus amyloliquefaciens*, *Aspergillus niger*, and *Trichoderma harzianum*, were artificially introduced into the pot experiment. This study investigated whether the artificial introduction of external microbial inoculants into granite rubble soil would impact the native soil microbial community and whether changes in this community would subsequently affect the root structure of tall fescue.

Similar to findings from other studies, the results of this experiment indicate that targeted microbial induction can alter the native microbial community structure in gravel soil [21]. Compared to the CK treatment, the number of bacterial and fungal OTUs in the granite rubble soil decreased to varying degrees after the application of microbial inoculants. The sequencing of fungal communities in the inoculated soils revealed that the K treatment exhibited the highest abundance of Basidiomycota at the phylum level, while Ascomycota was most abundant in the other treatments. The sequencing of the bacterial communities showed that Proteobacteria had the highest abundance in the CK, JD, H, and HC treatments, followed by Actinobacteriota. However, in the K treatment, Proteobacteria remained the most abundant, followed by Verrucomicrobiota. The application of microbial inoculants also affected the biomass of tall fescue, with the HC treatment significantly increasing the belowground biomass compared to the CK treatment; changes in the aboveground biomass were not significant. The root-to-shoot ratio, an indicator of plant adaptability to different environments [22], slightly increased following microbial inoculation, with the HC treatment showing a significant improvement over the CK treatment. As illustrated in Figure 5, the application of microbial inoculants promoted root development in tall fescue, leading to an increase in the number of lateral root branches. Notably, Pearson correlation analysis of the tall fescue root morphological traits revealed a negative correlation between the average root diameter and the other measured traits, suggesting that while an increase in the lateral roots can enhance the overall root surface area, it may lead to a reduction in the root diameter. The anatomical structure of the roots is closely related to the overall function of the plant. In this experiment, we measured the area of the main root cross-section and the stele diameter ratio in tall fescue, finding that the K, H, and HC treatments significantly affected the cross-sectional area of the main root, while the H treatment significantly influenced the stele sheath and cortical area ratios (*p* < 0.05). The JD treatment also had a significant impact on the cortical area ratio (*p* < 0.05).

Previous studies have demonstrated that plant roots can recruit different microorganisms through exudates, thereby shaping the structure of the rhizosphere microbial community [23]. Although the CK treatment exhibited higher OTU counts for both fungi and bacteria compared to the microbial inoculation treatments, it did not result in superior performance in terms of root biomass, root structure, or other plant growth metrics. While the application of microbial inoculants led to a reduction in OTU counts, it may have selectively promoted the emergence of dominant microbial populations that enhance root development. In other soil conditions, microbial inoculants typically have a significant positive effect on plant biomass; however, in this experiment, we did not observe such effects [21,24]. A possible explanation is that the nutrient-poor and low water retention characteristics of granite rubble soil prevent the applied microbial inoculants from sufficiently promoting plant growth and development, resulting in negligible changes in plant biomass [1]. The application of microbial inoculants in this study did not lead to root adaptation by reducing the number of root branches to promote elongation growth, which would reduce nutrient redundancy and waste; instead, it increased the number of lateral roots (Figure 5). An increase in the lateral roots can enhance soil stabilization by the plant roots, suggesting that the application of exogenous microorganisms in granite rubble soils may be useful in engineering fields to form root–soil complexes that enhance granite rubble soil stability [25]. Changes in the root anatomical structure represent a stable adaptive strategy developed by plants over long-term exposure to specific environments [3,26]. We observed that microorganisms influenced the cross-sectional anatomy of the main roots. The cortex, typically composed of parenchyma cells, plays a critical role in storage, transport, and gas exchange [3]. In this study, the application of microbial inoculants increased the cortical area ratio in the cross-sectional anatomy of tall fescue main roots, which may provide structural support and help maintain root morphology in gravel soil. Additionally, the increased cortical area may store more nutrients under adverse conditions, thus supporting the growth and development of tall fescue. The vascular bundles, consisting of xylem and phloem, did not show significant changes across treatments. In the H treatment, the epidermal area ratio of the main root cross-section was significantly lower compared to the CK treatment. The effect of *Aspergillus niger* on plants depends on the specific environment, plant species, and growth conditions of the fungus. Under proper control and utilization, *Aspergillus niger* can promote plant growth and protect plants from diseases, but under unfavorable conditions, it may have negative effects on plants [27,28]. The results of this experiment, where the tall fescue biomass and root structure were reduced under the *Aspergillus niger* treatment, suggest that in gravel soil conditions, *Aspergillus niger* may inhibit plant growth and is unlikely to support the growth and development of tall fescue.

**Figure 10:**
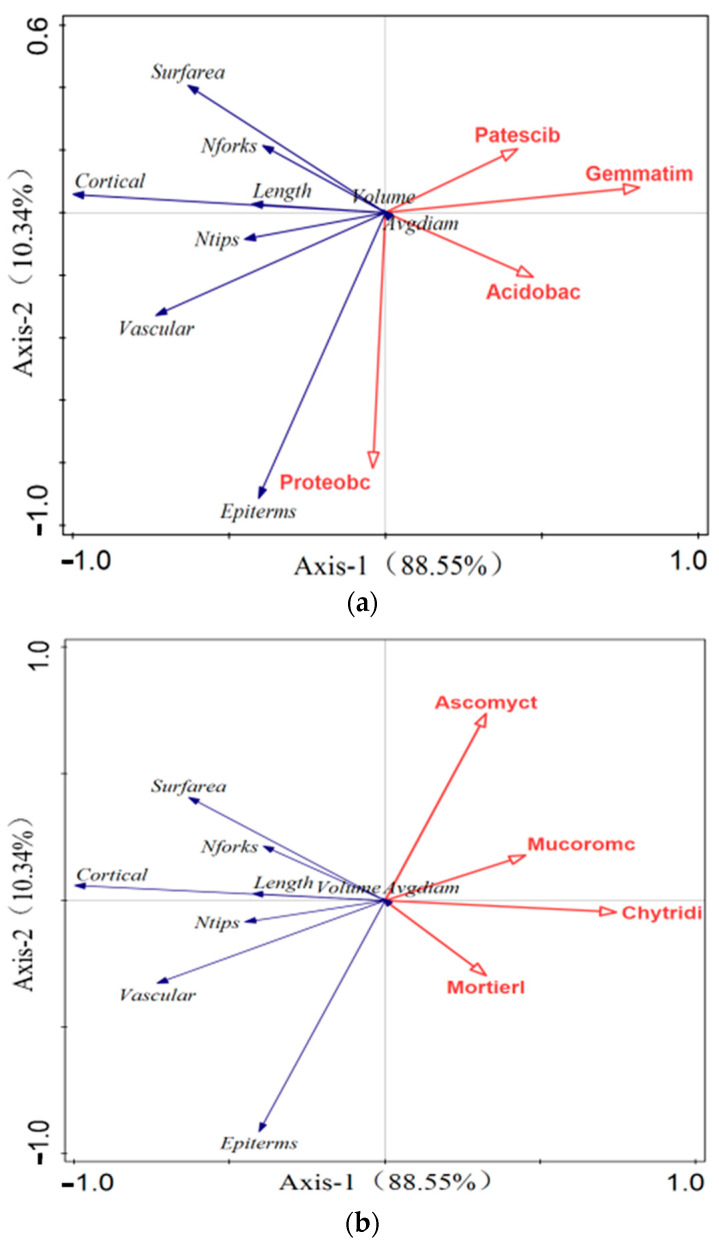
RDA analysis. (**a**) RDA analysis of the tall fescue root indicators and bacterial phyla. (**b**) RDA analysis of the tall fescue root indicators and fungal phyla.

To further investigate the effects of fungal and bacterial phyla on tall fescue roots, we performed a redundancy analysis (RDA), as shown in Figure 10. The RDA was conducted using tall fescue root traits, including the root length, root volume, root surface area, number of branches, number of crossings, root cortex area, root epidermis area, and root stele area, along with the four bacterial and fungal phyla with the greatest contributions. The analysis revealed that the bacterial phylum Proteobacteria was significantly correlated with the root cortex area, stele area, and the number of branches, playing a major role in influencing the root structure. The fungal phylum Mortierellomycota showed a strong correlation with the root epidermis area. The experiment demonstrated that the application of exogenous microorganisms can alter the phylum composition of the native soil microbial community, with interactions among different phyla influencing the root structure of tall fescue.

This experiment, conducted under pot conditions, provided an ideal experimental environment. In future studies, we plan to carry out field experiments to explore the effects of microbial inoculants on soil microbial communities and tall fescue growth in different environmental conditions. Additionally, this study focused solely on the impact of microbial community changes on the root structure of tall fescue. Future research should adopt a more integrated approach, analyzing the interactions among microbes, soil, and plants to further investigate their relationships, thereby providing both theoretical and practical support for the restoration of gravel soils. Moreover, we will continue to explore the effects of microbial root exudates on plant growth in gravel soils, aiming to expand the research in this field.

## 5. Conclusions

The following conclusions were drawn based on the plant–microbe interaction experiment:

1. The application of exogenous microorganisms changed the community diversity of fungi and bacteria in the plant root soil. After the fungal solution was applied, the dominant community of the root soil fungi and bacteria appeared under different treatments, and their abundance was lower than that of the blank control group. The relative abundance of microorganisms changed under each treatment, but the changes in the dominant population were not obvious.
2. Different microorganisms affected the biomass of tall fescue and increased the root–shoot ratio of the plant.
3. Changes in the microbial communities affected the conformation of the plant root system structure and the area share of the root system’s anatomical structure. The total root length, root surface area, and root volume of single plants were better than those in the blank control; the microorganisms affected the transverse area of the main roots of plants and increased the proportion of the transverse structural cortex under the bacterial addition treatments.
4. In the tall fescue root structure, the root cortex area, mid-column area, and number of branches were affected by *Proteobacteria*, while *Mortierellomycota* affected the root epidermal area.
5. Under granite rubble soil conditions, the application of *Aspergillus niger* exerted a more pronounced negative impact on tall fescue than positive effects. Consequently, the use of *Aspergillus niger* should be avoided in future applications.

## Figures and Tables

**Table 1:** Significance analysis of plant root morphology under different treatments.

Morphological Indicators	Treatment				
	CK	K	JD	H	HC
Total root length (cm)	137.46 ± 25.58 a	236.85 ± 75.16 a	141.31 ± 29.58 a	118.72 ± 63.18 a	144.78 ± 43.75 a
Root surface (cm^2^)	9.21 ± 1.37 a	13.35 ± 4.21 a	9.62 ± 2.18 a	7.25 ± 3.31 a	10.19 ± 2.17 a
Root volume (cm^3^)	0.05 ± 0.01 ab	0.07 ± 0.01 a	0.05 ± 0.01 ab	0.03 ± 0.01 b	0.05 ± 0.01 ab
Average diameter (mm)	0.217 ± 0.025 a	0.220 ± 0.030 a	0.215 ± 0.009 a	0.207 ± 0.032 a	0.231 ± 0.021 a
Root tip (No.)	1888 ± 350 a	2771 ± 1501 a	1820 ± 365 a	1583 ± 773 a	2508 ± 266 a
Root forks (No.)	1001 ± 249 a	1540 ± 961 a	1161 ± 292 a	1039 ± 663 a	1309 ± 388 a

Note: Different lowercase letters represent significant differences (*p* < 0.05) among the treatments for the same indicator, *n* = 5. CK is the blank control; K is the *Bacillus subtilis* application; JD is the *Bacillus amyloliquefaciens* application; H is the *Aspergillus niger* application; and HC is the *Trichoderma harzianum* application.

**Table 2:** Pearson analysis of plant root morphology.

Morphological Indicators	Total Root Length (cm)	Root Surface (cm^2^)	Root Volume (cm^3^)	Average Diameter (mm)	Root Tip (No.)	Root Forks (No.)
Total root length	1					
Root surface	0.959 **	1				
Root volume	0.836 **	0.928 **	1			
Average diameter	−532 *	−0.424	−0.088	1		
Root tip	0.865 **	0.901 **	0.763 **	−0.508	1	
Root forks	0.899 **	0.926 **	0.747 **	−0.627 *	0.930 **	1

Note: * At the 0.05 level (two-tailed), the correlation is significant. ** At the 0.01 level (two-tailed), the correlation is significant.

**Table 3:** Basic physical and chemical properties of experimental granite rubble soil.

Indicators	Moisture Content (%)	pH	Organic Substance (g/kg)	Ammonium Nitrogen (mg/kg)	Quick-Acting Phosphorus (mg/kg)	Quick-Acting Potassium (mg/kg)
Results	9.83 ± 0.96	8.34 ± 0.05	0.42 ± 0.05	23.04 ± 0.62	7.50 ± 0.44	25.47 ± 0.91

Note: Data are expressed as the mean ± standard deviation.

## Data Availability

The original contributions presented in the study are included in the article. Further inquiries can be directed to the corresponding author.
